# Online proton therapy monitoring: clinical test of a Silicon-photodetector-based in-beam PET

**DOI:** 10.1038/s41598-018-22325-6

**Published:** 2018-03-06

**Authors:** Veronica Ferrero, Elisa Fiorina, Matteo Morrocchi, Francesco Pennazio, Guido Baroni, Giuseppe Battistoni, Nicola Belcari, Niccolo’ Camarlinghi, Mario Ciocca, Alberto Del Guerra, Marco Donetti, Simona Giordanengo, Giuseppe Giraudo, Vincenzo Patera, Cristiana Peroni, Angelo Rivetti, Manuel Dionisio da Rocha Rolo, Sandro Rossi, Valeria Rosso, Giancarlo Sportelli, Sara Tampellini, Francesca Valvo, Richard Wheadon, Piergiorgio Cerello, Maria Giuseppina Bisogni

**Affiliations:** 1 INFN, Sezione di Torino, Torino, Italy; 2 Department of Physics, University of Torino, Torino, Italy; 3 Department of Physics, University of Pisa, Pisa, Italy; 4 INFN, Sezione di Pisa, Pisa, Italy; 5 Dipartimento di Elettronica, Informazione e Bioingegneria, Politecnico di Milano, Milano, Italy; 6 INFN, Sezione di Milano, Milano, Italy; 7 Fondazione CNAO, Pavia, Italy; 8 Dipartimento di Scienze di Base e Applicate per l’Ingegneria, University of Roma ‘La Sapienza’, La Sapienza, Italy; 9 INFN, Sezione di Roma 1, Rome, Italy

## Abstract

Particle therapy exploits the energy deposition pattern of hadron beams. The narrow Bragg Peak at the end of range is a major advantage but range uncertainties can cause severe damage and require online verification to maximise the effectiveness in clinics. In-beam Positron Emission Tomography (PET) is a non-invasive, promising *in-vivo* technique, which consists in the measurement of the β+ activity induced by beam-tissue interactions during treatment, and presents the highest correlation of the measured activity distribution with the deposited dose, since it is not much influenced by biological washout. Here we report the first clinical results obtained with a state-of-the-art in-beam PET scanner, with on-the-fly reconstruction of the activity distribution during irradiation. An automated time-resolved quantitative analysis was tested on a lacrimal gland carcinoma case, monitored during two consecutive treatment sessions. The 3D activity map was reconstructed every 10 s, with an average delay between beam delivery and image availability of about 6 s. The correlation coefficient of 3D activity maps for the two sessions (above 0.9 after 120 s) and the range agreement (within 1 mm) prove the suitability of in-beam PET for online range verification during treatment, a crucial step towards adaptive strategies in particle therapy.

## Introduction

Particle therapy, one of the most promising cancer therapy techniques, is based on the dose delivered by charged particles, such as protons or heavier ions, which can be shaped in narrow beams. Their main advantage lies in their energy deposition pattern in matter, which exhibits the so-called Bragg Peak (BP) at the end of range, where the majority of the energy is released. Since the BP is very narrow, range uncertainties, due for example to dose calculation approximations in the treatment planning or to patient mispositioning and/or anatomical modifications at the time of irradiation, could cause severe damage to the patient, especially for tumours very close to critical organs. Presently, clinical facilities design treatment plans based on relatively large margins (up to 15 mm, depending on the particle range) and multi-directional delivery, so as to minimise the risk of critical damage. The implementation of reliable online range verification methods could allow both reduction of safety margins and dose escalation, leading to a full exploitation of the advantages of particle therapy in clinics. In-beam, off-beam or after-treatment Positron Emission Tomography (PET), secondary charged particles tracking, and prompt photon monitoring techniques have already been exploited clinically by various centres^1–7^. In particular, PET-based monitoring techniques rely on positron-emitting (β+) radioactive isotopes, which are produced by the target and, in the case of ions with Z ≥ 5, also by the projectile nuclear fragmentation^8^. The low-energy positrons emitted annihilate at rest in the body tissues producing 511 keV back-to-back photon pairs, a signal that can be exploited to monitor the treatment with a PET scanner^9^. Although the correlation between the measured activity and delivered dose is not straightforward^1,10^, the reconstructed activity distribution gives an insight into the coherence of the planned and delivered treatment.

In-beam PET, not being much influenced by biological washout associated to the metabolism, such as perfusion and diffusion through functional pathways, presents the highest correlation of the measured activity distribution with the deposited dose^11^; however, its past clinical implementations^6,12^ relied on sub-optimal instrumentation^2^. Here we report the first clinical results obtained with a state-of-the-art in-beam PET scanner, based on solid-state photodetectors and custom front-end electronics, time-resolved analysis and an on-the-fly reconstruction of the activity distribution during irradiation.

The INSIDE (Innovative Solutions for In-beam DosimEtry in hadrontherapy) in-beam PET scanner features two planar heads of 10 × 25 cm^2^ active area, each one made of 2 × 5 detection modules with 16 × 16 Lutetium Fine Silicate (LFS) crystals coupled 1:1 to Hamamatsu MPPCs, resulting in 2560^2^ lines of response. The PET heads are positioned above and below the patient, at a relative distance of 60 cm; therefore the Field Of View (FOV) covers 25 cm along the beam direction, 10 and 22 cm along the transverse and vertical directions, respectively. The detector resolution on the photon interaction point in the crystal is in the order of the mm, thans to the 1-to-1 coupling between the crystals (3.2 mm pitch) and the SiPM.

The TOFPET ASIC^13^ is used to read out information about the energy and time of each event. The events are processed by 20 Xilinx Spartan-6 Field Programmable Gate Arrays and transmitted with UDP protocol through Gigabit Ethernet links to a HP Proliant data acquisition server which implements the online coincidence finding and reconstructs the PET images on-the-fly, in about 6 s. The scanner is mounted on a mobile support and the distance between the PET heads can be adjusted to reduce possible interference with the treatment room equipment. A detailed system description is available in^14^.

The scanner has been installed in one of the Italian National Center of Oncological Hadrontherapy (CNAO)^15^ synchrotron facility treatment rooms with a fixed horizontal beam line. The in-beam PET was tested during 2016 with polymethyl methacrylate (PMMA) and anthropomorphic phantoms^14,16^ and its response was thoroughly characterized.

**Figure 1:**
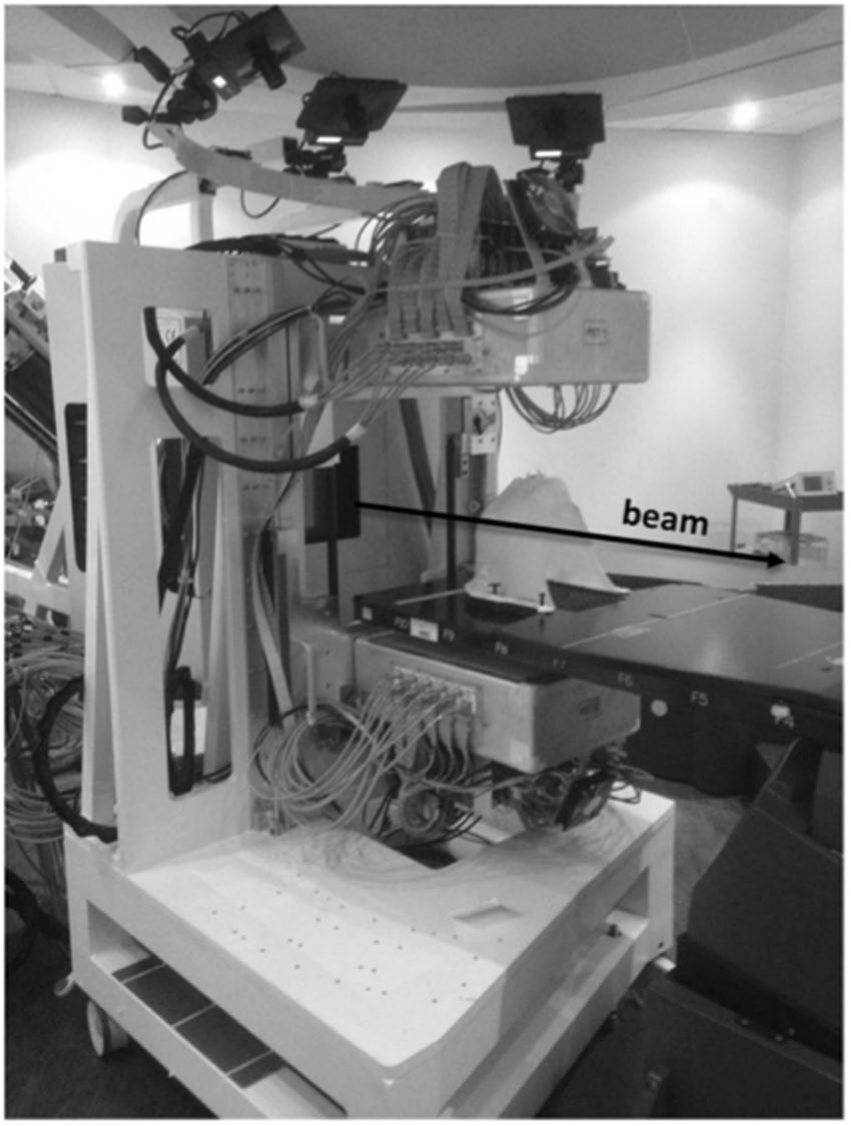
The INSIDE in-beam PET in one of the CNAO treatment rooms. The mobile support is placed between the horizontal beam line nozzle and the patient bed, ready for acquisition. The beam direction is shown in the picture.

The scanner setup used during system characterization was adopted on December, 1^st^ and 2^nd^ 2016 to perform the first online monitoring test during a patient treatment session. The scanner support was positioned between the beam nozzle and the patient bed, by manual alignment with the treatment room lasers (Fig. 1). In order to avoid interference with patient handling and X-ray patient position verification, the scanner was manually put in place just before the field delivery and removed shortly afterwards.

**Figure 2:**
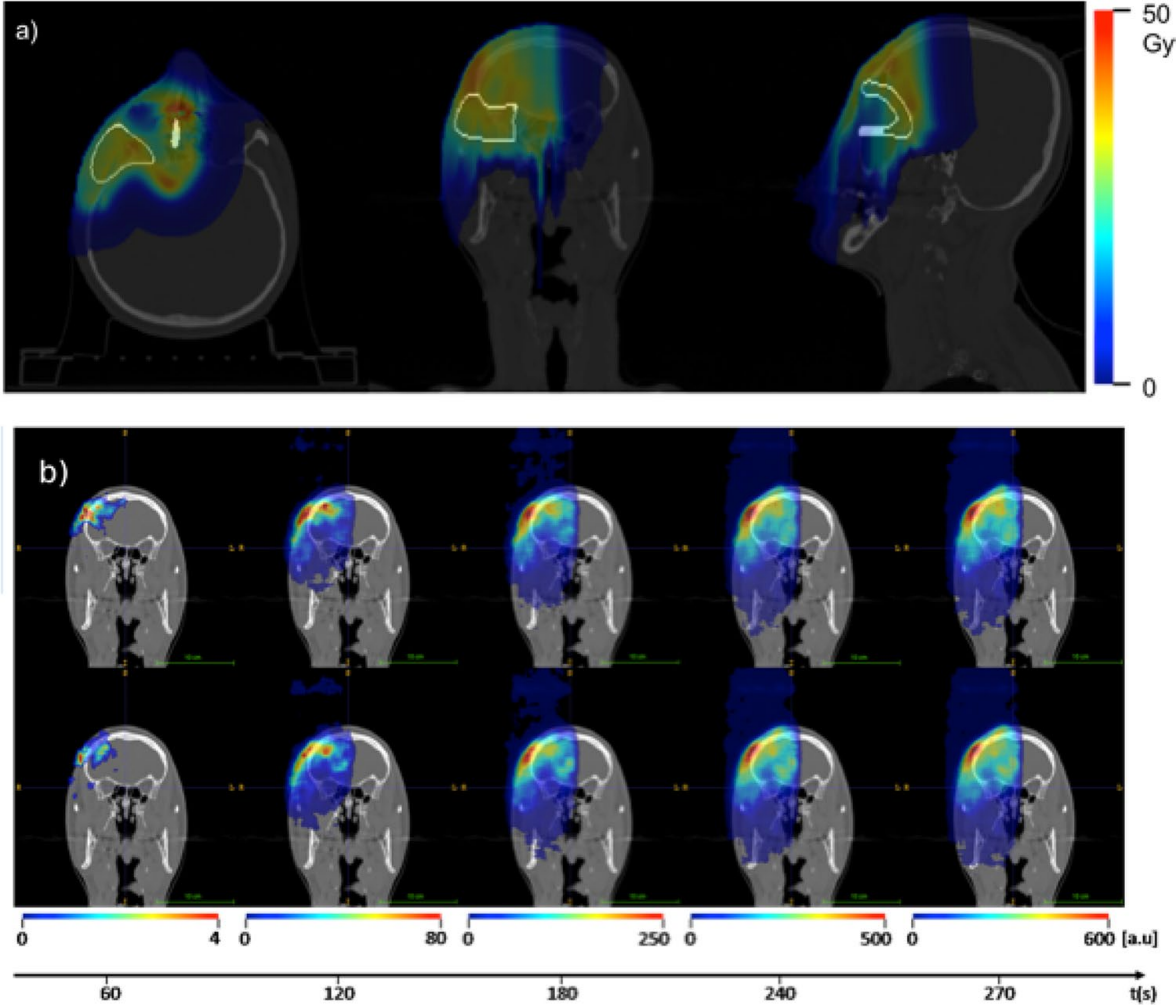
(**a**) Treatment plan and set up. Axial (left), coronal (centre) and sagittal (right) sections of the patient CT with the planned dose distribution to be delivered in the beam field monitored with the INSIDE in-beam PET system and the Clinical Target Volume (CTV) superimposed in white. (**b**) Time Evolution of a 2D slice of the detected beam-induced activity superimposed to the patient Computed Ttomography (CT) used for dose planning. The top and bottom rows refer to the first (December, 1st, 2016), and second (December, 2nd, 2016) acquisition days, respectively. The shown images correspond to 3D activity map reconstructions at the end of every minute, starting from the beginning of the treatment. An additional image corresponding to the whole treatment plus 30 s after-treatment is also shown. The image look-up tables refer to different intensity scales because of the different amounts of data integrated during the time intervals.

The patient (male, 56 years old), affected by carcinoma of the right lacrimal gland, was treated with protons in 30 daily sessions of 2.2 GyE each. The treatment was delivered with a fixed horizontal beam line (IEC Gantry Angle = 90°), with two beam fields. The INSIDE in-beam PET system was tested with the first irradiated beam field, roughly corresponding to a half session dose. Figure 2a shows the planned dose distribution to be delivered during the monitored field, superimposed to the patient’s CT. The clinical target volume (CTV) is also shown.

On the days of the INSIDE in-beam PET acquisition, the patient underwent fractions n. 28 and 29 of his therapy. In-beam PET acquisition was performed during the field irradiation with the patient bed positioned parallel to the beam axis (IEC Patient Support Angle equal to 270°). The PET heads were placed as in Fig. 1. The distance of the beam isocenter from the upper and lower detector active surface was 32.7 cm and 27.6 cm, respectively. The configuration was not symmetric because of mechanical constraints related to the positioning of the patient bed.

The aim of the present analysis is to demonstrate the feasibility of online monitoring with PET in a clinical environment and the reproducibility of the measurements. Therefore, the analysis is carried on comparing the activity acquired in the two treatment sessions.

## Results

### On-the-fly reconstruction

In the measured beam field, 3.7∙10^10^ protons were delivered with an energy range from 66.3 to 144.4 MeV, divided in 56 pencil-beam scanning slices, with a 3.12 cm water-equivalent thick range shifter positioned along the beam axis, 11 cm from the isocenter. The irradiated volume maximum cross dimension with respect to the beam axis was about 10.6 × 10.0 cm^2^. The duration of the beam field irradiation was about 240 s; the in-beam PET acquisition lasted 30 s more, corresponding to the time necessary to access the room, remove the scanner and start preparing for the administration of the second field.

For both acquisitions, the PET images were reconstructed on-the-fly every 10 s from the beginning of the treatment, as seen in the video (included as supplementary information) that compares the two treatment sessions, so as to analyse the time evolution of the activity distribution while progressively integrating the signal and prove the online monitoring feasibility. The reconstructed images were available for analysis with a delay of about 6 s, (corresponding to about 1.5 accelerator cycles – see section 4.1), which would allow treatment interruption in case a range error was detected. The beam is delivered with increasing energy, in periods of actual particle delivery (*in-spill*) of about 1 s, followed by intervals (*inter-spill*) of about 3 s. As explained in the *Methods* section, the reconstruction was made taking into account only the coincidences acquired during the *inter-spill* phase. A final image including the whole treatment and 30 s after-treatment was also reconstructed.

Figure 2b shows some of the images reconstructed during treatment, as a function of the acquisition time (every 60 s), superimposed with the patient Computed Tomography (CT) used for dose planning. A median filter with a 11.2 × 11.2 × 11.2 mm^3^ kernel (7 × 7 × 7 voxels) was applied to the in-beam PET images in order to minimise low statistics and shot noise biases.

The shape variations of 3D activity images reconstructed up to about 60 s, related to the collected statistics, are clearly visible between the two acquisitions. As the treatment progresses, the integrated activity increases and the images series are more and more similar, as confirmed by the quantitative evaluation.

**Figure 3:**
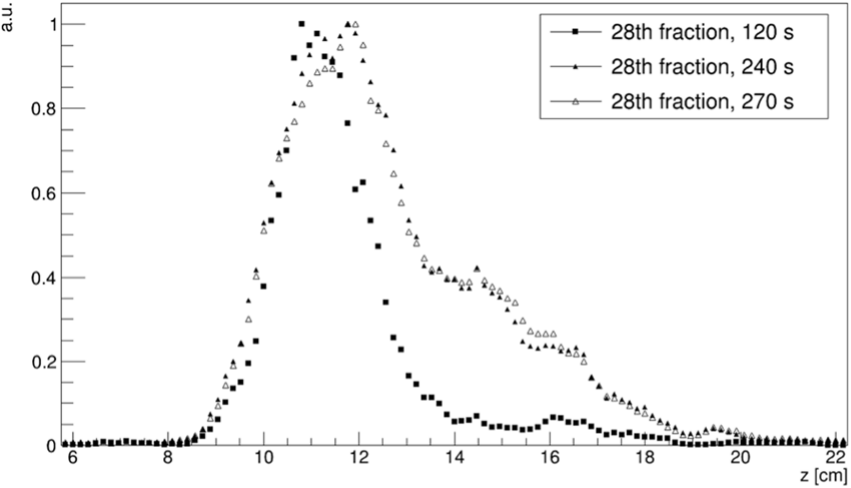
Reconstructed activity profiles along the beam direction (z) for 1 pixel in the transverse plane (xy), at three different time intervals corresponding to one half of the delivery (120 s), the end of treatment (240 s) and the end of acquisition (270 s). The distributions are normalised to their maximum activity value.

As an example, the activity profiles along the beam direction (z) for three different time intervals are shown in Fig. 3. The profiles, calculated by projecting along the beam axis a 1.6 × 1.6 mm^2^ area (1 × 1 voxel) in the transverse plane (xy), clearly show that the range progresses with time (as expected, since the beam energy increases during the delivery) and that the fall-off, although spread over a few cm, is smooth enough to allow a comparison between different treatment sessions.

### Time-resolved analysis

In this work, three analyses – described in detail in section 4 – were implemented, labelled as Pearson’s correlation coefficient (PCC), Beam’s Eye View (BEV) and Overall View (OV). All of them take into account only the image volume actually irradiated by the beam. The results obtained on the time-resolved image series acquired in the two consecutive days provide a first evaluation of the INSIDE in-beam PET performance, which will be studied in depth in the future with the evaluation of a wide range of patients.

**Figure 4:**
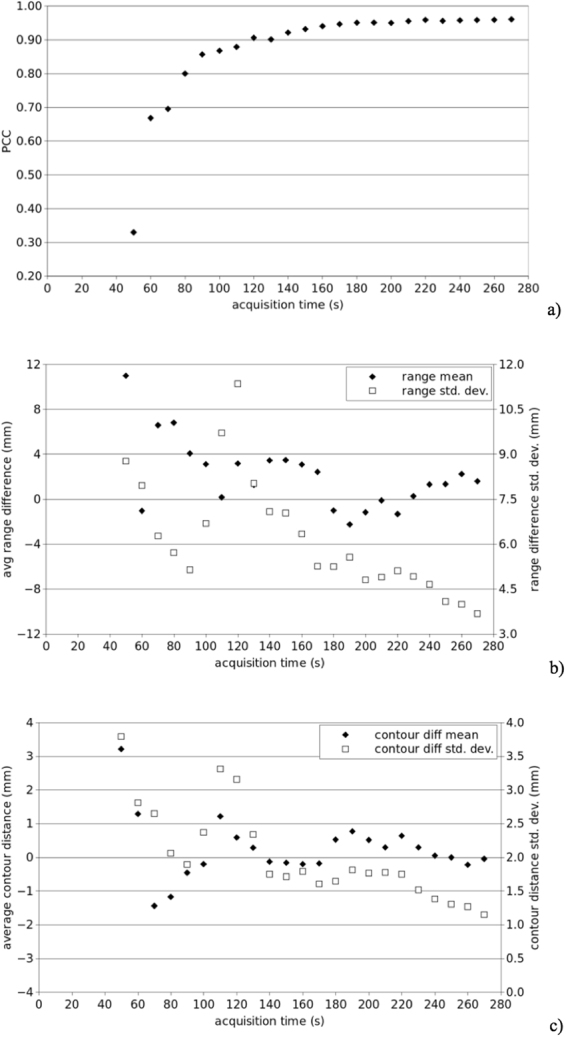
(**a**) Pearson’s Correlation Coefficient calculated for each couple of PET images, reconstructed every 10 s, as a function of time. (**b**) Mean difference (black) and standard deviation (white) calculated with the BEV method. (**c**) Mean difference (black) and standard deviation (white) calculated with the OV method.

The PCC analysis was implemented to obtain an overall index quantifying the linear correlation between the intensity of the image series from the two-days. The PCC index is not sufficient to rule out local range anomalies, which are better addressed with the BEV and OEV methods, but it provides a fairly simple first order evaluation of the compliance between the expected and actual activity distributions. The PCC was computed in a Region Of Interest (ROI), defined in detail in the *Methods* section, that includes the activity distal fall-off inside the patient body^17^. Figure 4a shows the PCC evolution as a function of time. After 120 s the PCC reaches values above 0.90, showing good agreement between acquisitions, and its maximum (0.96) corresponds to the last couple of acquired images.

Since the main goal of beam monitoring is to assess *in-vivo* the particle range, the BEV method analyses the 3D activity distribution taking into account the direction of the beam (Fig. 4b). This method evaluates the differences in the activity range between two PET images along the beam axis. After 120 s, the average range difference is in the interval (−2.2, +3.5) mm. The BEV method is specifically prone to two uncertainty sources: low statistics, which results in reduced sensitivity, and repeatability of the scanner positioning on the two acquisitions (presently, about 1–2 mm on the horizontal plane).

The OV method, on the other hand, circumvents these uncertainty sources: it compares images without any preferred direction, by finding the minimum distance point-by-point of the two contour surfaces. Results after 120 s (Fig. 4c) indicate sub-millimetric agreement of the average surface distance until the end of treatment.

The BEV and OV methods do not provide reproducible information until the signal acquisition time is longer than 120 s because the integrated activity is not statistically significant. Further analysis will be conducted to evaluate the feasibility and advantages given by PET images reconstructed with integration times shorter than 60 s.

## Discussion

Until today, the most significant clinical implementation of in-beam PET was at the GSI synchrotron Helmholtz Centre for Heavy Ion Research in Darmstadt, Germany, between 1997 and 2008^12^, to monitor carbon ion therapy. Among the many important contributions of this study is the significant improvement of the model used to convert CT HU map into human tissues and densities^18^. Another installation is presently in clinical use at HIMAC in Kashiwa, Japan^9^; it is well integrated within the cyclotron gantry, but it acquires data only after the irradiation. Several other systems are under construction or being commissioned^6,19^.

The INSIDE scanner presented here relies on state-of-the-art technologies, and is the first, to our knowledge, able to demonstrate that in-beam PET imaging can be effectively used to monitor proton therapy in clinical conditions, while delivering the treatment. Once the statistical significance required for a meaningful analysis is reached (about 2 minutes after the beam delivery started), the online feedback on the treatment compliance is available with a delay of 6 s, enough to implement emergency procedures to prevent unexpected damage to the patients.

Moreover, an automated time-resolved analysis was proposed, which is a fundamental goal to achieve on-the-fly quantitative treatment verification. In literature, the evaluation of the treatment accuracy by means of PET images has already been investigated with different strategies. Most of the published works focused on the evaluation of the range considering the distal activity fall-off^6,20–22^, or otherwise relied on visual analysis^5^. Among them, quantitative approaches aim to determine the activity range inside the patient body by thresholding or minimization/optimization techniques^23^. Our automated time-resolved analysis shows a strong correlation between the two image series (PCC > 0.9, and average surface distance < 1 mm after 120 s). This result is in accordance to the expected outcome, since the acquisitions refer to two consecutive treatment days, and no relevant anatomical difference was expected, but it also demonstrates that, after about half treatment, the in-beam PET signal is already significant and can be used to detect abnormalities.

After the successful first *in-vivo* test, the scanner mechanical support is being revised in order to integrate it more smoothly in the clinical workflow. The primary reason is to shorten the positioning time and hence avoid unnecessary extension of the patient immobilization time; also, the new holder will lead to a more accurate positioning (of the order of 1 mm, while presently the uncertainty in the horizontal plane is about 2 mm). The revised mechanics will avoid the interference with the bed positioning and will allow improvements in the results in terms of statistical significance (by extending the number of studied patients and fractions), and accuracy, for both proton and carbon ion therapy.

The simulation of the 3D-activity distribution induced by the delivered treatment^24,25^ is being refined, and it will be used as prior knowledge and serve as a reference for comparison with the experimental measurements at the beginning of the treatment cycle^12^.

A clinical trial focused on evaluating the INSIDE performance on a cohort of patients will start in 2018. The parameters used in the PCC, BEV and OV methods will be optimised and the range variations for different treatment plans, along their longituinal evolution, will be measured.

With the new mechanical layout, the clinical workflow will start with the patient positioning procedure, followed by the placement of the INSIDE scanner and the treatment delivery. The limitations related to the sub-otpimal spatial resolution on the vertical coordinate will be addressed by integrating the time-of-flight information in the reconstruction. An optimisation of the front-end electronics time resolution will also help reducing the background caused by prompt photons random coincidences in the energy range of the photopeak and could allow the use of data taken during the spill delivery to improve the statistics and shorten the time needed to obtain the first reliable 3D activity map.

## Methods

The clinical study was performed at the CNAO facility (Pavia, Italy) in accordance with relevant guidelines and regulations and was approved by the CNAO ethics committee (request number 20170011630); the informed consent was obtained from the participant. No information or images that could lead to identification of the participant is present in this work.

The datasets generated and analysed during the current study are available from the corresponding author on reasonable request.

### Data selection and image reconstruction

The CNAO synchrotron beam delivery is conducted using an intensity-controlled raster scanning technique, with increasing energy and a characteristic time structure defined by about 1s-long time intervals in which the particles bunches are actually delivered (called *in-spill*), and about 3s-long time intervals where the beam is off (*inter-spill*)^26^. During the tests with phantoms aimed at the scanner characterization^27,28^, the INSIDE in-beam PET system efficiently acquired in list mode and reconstructed data in both *in-* and *inter-spill* conditions.

In-spill is dominated by prompt photons and neutron radiation and the single event rate detected during the clinical acquisition was higher than inter-spill by almost a factor 30. Data quality is inherently lower during in-spill, since pair production is originated also by prompt radiation, which is less correlated to the irradiated volume with respect to **β**^**+**^ decay. Hence coincidence data was selected from inter-spill only by applying a threshold on the acquisition rate. About 7.6 ∙ 10^4^ coincidences were left for the activity image reconstruction after a 270s-long acquisition time interval.

A maximum likelihood expectation maximization (MLEM) algorithm with 5 iterations was used to reconstruct time-resolved activity images^29^. Reconstruction time takes about 2 to 5 seconds. The attenuation correction was applied, using the CT acquired for treatment^30^.

### Analysis methods

All the analysis methods reported in this section were implemented in C++ with ITK libraries [www.itk.org].

#### Pearson Correlation Coefficient (PCC)

The PCC was evaluated by calculating the correlation coefficient in a Region Of Interest (ROI) that includes the activity distal fall-off^17^. Due to the complex geometrical shape of the treated volume, the ROI was defined by considering the voxels between the first voxel with intensity larger than 95% and the last voxel with intensity larger than 25% of the maximum value of the profile along the beam direction. In the ROI definition, only the irradiated volume (i.e., the volume corresponding to the surface through which the beam passed during the treatment) was taken into account. Since the selected ROI in the two PET images must be the same in order to calculate the PCC, the logic union of the individual custom ROIs was used.

#### Activity mask extraction for the BEV and OV methods

In order to remove the background noise and identify the activity range inside the patient body, the 3D activity distribution was analysed as follows. An activity mask is extracted by applying a threshold filter tunable to the maximum activity intensity of each image (set to 10% in the present analysis), coupled to erosion and dilation filters to obtain a fully connected volume. Because of the low acceptance angle of the in-beam PET scanner planar geometry, a poor resolution in the reconstruction of the activity position along the direction between the two PET heads was expected. To overcome this intrinsic limitation and in agreement with procedures reported in literature^1,5,6^, the difference in activity range in BEV and OV methods was evaluated solely in the volume in which the proton beam was actually delivered, by using the time-dependent information given by the dose delivery system.

#### Beam’s eye view (BEV)

The Beam’s eye view method aims to define the activity range from the beam’s point of view, and, therefore, takes into account in the image only the actually irradiated volume. The starting and ending point of the activity range are calculated, for each voxel belonging to the transversal plane with respect to the beam direction, by analysing the activity mask contour. The activity range values for the two treatment sessions were then compared by evaluating the average and standard deviation of their difference. Thanks to the INSIDE in-beam PET system capability to obtain time-resolved image series, the BEV analysis was performed for each image reconstructed at different time intervals, in order to study the time evolution of the range difference indicators. The observables are not influenced by shot noise thanks to the robustness of the procedure for the activity mask extraction. They are instead sensitive to shifts of the PET scanner working position, which is presently settled manually.

#### Overall view (OV)

To partially overcome uncertainties due to the positioning procedure of the mechanical support, which did not allow a precise alignment of the detector with the beam, an analysis independent of the beam direction was implemented. In the OV analysis, the activity mask contours were used to compare in time the images acquired in the two consecutive sessions, considering only data referring to the actual irradiated region (same as in the BEV method). The fundamental difference from the previous method is that the contour comparison takes into account every 3D possible direction to determine the minimum Euclidean distance, voxel by voxel, between contours. The average and standard deviation of the contour differences are expected to be smaller than the range difference indicators obtained with the BEV method because the OV analysis is less sensitive to tilts or translations in the detector working position between the two acquisition sessions. Thanks to the activity mask extraction procedure, this method is also weakly influenced by shot noise.

## Electronic supplementary material

Supplementary Information
